# Identification and Candidate Gene Analysis of *Brcl1*, a Novel Gene Confers a Leaf Curled Phenotype in *Brassica rapa* L.

**DOI:** 10.3390/ijms26020732

**Published:** 2025-01-16

**Authors:** Lihui Wang, Huishan Liu, Yunxia Sun, Wei Wang, Chao Li, Yuanwei Liu, Zhiyong Liu, Ruiqin Ji, Shengnan Huang, Gaoyang Qu, Yugang Wang

**Affiliations:** 1 Department of Horticulture, Shenyang Agricultural University, Shenyang 110866, China; 2 Department of Horticulture, Hunan Agricultural University, Changsha 410128, China

**Keywords:** leaf curl, *Brassica rapa*, EMS, fine mapping, *RGL1*

## Abstract

Leaf shape is an important determinant of photosynthesis, yield and quality in plants. In this study, we obtained a curled leaf mutant, *cl1*, from an ethyl methanesulfonate (EMS)-induced mutagenesis population. It was designated the *Brcl1*^YS^ locus. Bulk segregant RNA sequencing combined with recombinant screening identified the candidate interval responsible for *Brcl1*^YS^ in a 97.5 kb region on chromosome A02. Twelve genes were identified within the candidate region. Sequence differences and co-separation verification confirmed that *BraA02g017030.3C* was the most promising candidate gene underlying the *Brcl1*^YS^ locus. It is homologous to *Arabidopsis AT1G66350* (*RGL1*), which has been shown to act as a negative regulator of the gibberellin pathway. Combined with cell morphology observation, it is speculated that the loss of function of *Brcl1*^YS^ results in differences in cell development, ultimately leading to changes in leaf morphology. The results will contribute to the understanding of the molecular mechanisms underlying leaf curling in *B*. *rapa*.

## 1. Introduction

*Brassica rapa* is cultivated worldwide as an important crop, primarily for its leafy vegetables, such as Chinese cabbage and Pak-choi. It is also grown partly for fodder and oilseed production, including turnip rape and Yellow Sarson [1]. Leaf shape reflects a number of trade-offs, including light capture, gas exchange and temperature buffering [2]. Light absorption is the main driver of photosynthesis in domesticated plants and is one of the main determinants of crop yield [3,4,5]. Moderate leaf curling can enhance plant resilience by increasing light absorption and reducing water loss under adverse conditions [6,7]. This contributes to improved photosynthetic efficiency and grain yield. Thus, the molecular basis of leaf curling could provide a theoretical basis for growth and yield in *B. rapa*.

Leaf shape is influenced by various factors, including the external environment, internal gene expression levels, and hormone levels [8,9]. Several genes have been reported to be associated with leaf development, including *KANADI*, *Class III HOMEODOMAIN LEUCINE-ZIPPER* (*HD*-*Zip*-*III*), *WUSCHEL RELATED HOMEOBOX* (*WOX*), *TB1*-*CYC*-*PCFs* (*TCPs*) and other transcription factors involved in the establishment of leaf polarity [10,11,12,13]. It was also shown that hormonal factors are important in the process of leaf development. The uneven distribution of auxin (IAA) in the plant can cause leaf curling [14]. Meanwhile, the mutation of *AUXIN/INDOLE-3-ACETIC ACID* (*Aux/IAA*) gene family could cause leaf curling and dwarfism in *Arabidopsis*, *Brassica napus*, and *Vaccinium uliginosum* L. [15,16,17]. *AUXIN RESPONSE FACTORS* (*ARFs*) could interact with *HD-ZIP IIIs* and *KANADI* to regulate leaf development, and the loss of *ARF3* function in *Arabidopsis* lead to leaf curling [18]. These studies have shown that deficient expression of genes encoding proteins that mediate phytohormone biosynthesis or signaling are responsible for abnormalities in some leaf flatness traits [19,20]. However, as a significant hormone regulating plant growth, the role of gibberellin (GA) in leaf curling has not been reported.

GA was involved in almost the whole process of plant growth, including seed germination, flowering and fruit development [21]. Through mass genetic screening and analysis in various plants, DELLA proteins act as key oppressors in the GA signaling pathway, which belongs to the *GRAS* gene family and includes *GAI* (*GA-insensitive*), *RGA* (*Repressor of ga1-3*), *RGL1* (*RGA-like1*), *RGL2* (*RGA-like2*) and *RGL3* (*RGA-like3*) [22,23]. DELLA proteins are highly influential, as they can interact with many hormones and environmental response factors to regulate plant growth and development [24]. Recent reports found that DELLA proteins positively regulate seed size by enhancing cell division in *Arabidopsis* [25], and they can interact with NAC though the GA signaling to promote secondary cell wall formation in cotton [26]. As a member of the DELLA protein subfamily, over expression of *RGL1* decreased the content of GA and regulated seed germination, leaf expansion, flowering, stem elongation and flower development [27]. In maize, changes in local GA level could regulate the leaf morphology by affecting cell division [28]. Therefore, *RGL1* may regulate the content of hormones or cell division to control leaf morphology.

In the present study, we identified an EMS mutant, *Brcl1*^YS^, which was phenotypically characterized by significant leaf curling compared to the recipient parent, Yellow Sarson (YS). We fine-mapped the locus responsible for the leaf curling trait using single nucleotide polymorphisms (SNPs) and other molecular markers. We identified the candidate gene for the trait based on gene sequencing, candidate gene co-separation and expression analyses. Our results will provide clues for further positional cloning and functional research on *Brcl1*^YS^ and provide a good basis for elucidating the molecular mechanism underlying the leaf curl trait in *B. rapa*.

## 2. Results

### 2.1. Characteristics of Brcl1^YS^

**Figure 1:**
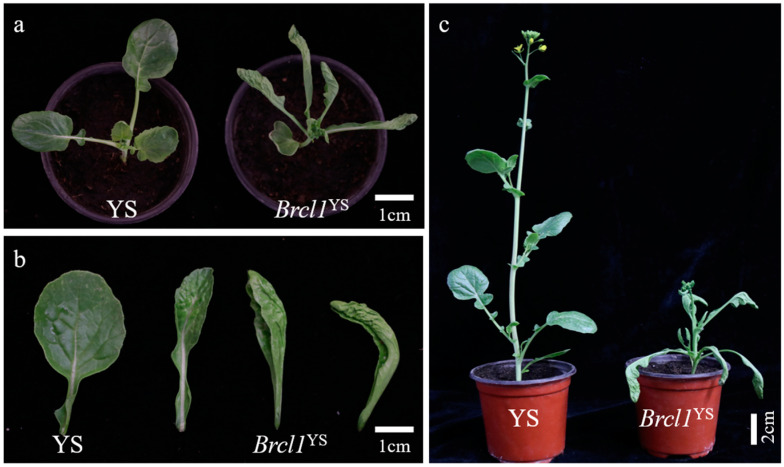
Phenotypic characterization of YS and *Brcl1*^YS^. (**a**) The orthograph of YS and *Brcl1*^YS^, scale bars = 1 cm; (**b**) the leaf of YS and leaves at different period of *Brcl1*^YS^, scale bars = 1 cm; (**c**) the flowering stage of YS and *Brcl1*^YS^, scale bars = 2 cm.

Each ten individuals, from 15 to 50 days, in the similar growth stage, were utilized to detect variations between the two parents, *Brcl1*^YS^ and YS. Compared with the YS, the leaves of *Brcl1*^YS^ were up-curled and crinkled, whereas YS had normal, flat leaves (Figure 1a,b). In addition, the *Brcl1*^YS^ had shorter stature (Figure 1c). The plant height, stem width, leaf length and leaf width were further determined after 45 days. All the four indicators, including plant height (YS with 8.97 ± 0.76 cm and *Brcl1*^YS^ with 4.98 ± 0.44 cm), stem width (YS with 0.74 ± 0.02 cm and *Brcl1*^YS^ with 0.62 ± 0.06 cm), leaf length (YS with 8.38 ± 0.53 cm and *Brcl1*^YS^ with 6.13 ± 0.26 cm), and leaf width (YS with 5.38 ± 0.22 cm and *Brcl1*^YS^ with 3.35 ± 0.19 cm), showed significant differences between the two parents, YS and *Brcl1*^YS^ (Table 1).

### 2.2. Genetic Analysis of Curled Leaf in Mutant Brcl1^YS^

The phenotype of both reciprocal F_1_ showed no difference with the curled parent, *Brcl1*^YS^. There were no significant differences observed between the F_1_ and reciprocal F_1_ individuals, and both of them showed curled leaves, similar to the curled parent, *Brcl1*^YS^.

The segregation ratio of the subsequent F_2_ population was in accordance with the expected Mendelian segregation ratio of 3:1 (Curled vs. Normal leaves) (Table 2). It can be concluded that the curled leaf was controlled by a pair of dominant genes in *B. rapa*.

### 2.3. Analysis of Physiological Indices

Our study evaluated the photosynthetic rate (Pn), the intercellular CO_2_ concentration (Ci), the transpiration rate (E), stomatal conductance (Gs) and the photosynthetic pigment content (chlorophyll a, chlorophyll b, chlorophyll, and carotenoid) to identify the difference between *Brcl1*^YS^ and YS (Table 3). Compared with YS, the Pn of *Brcl1*^YS^ was significantly increased, while the E, Ci and Gs were significantly decreased. Additionally, the photosynthetic pigment content of *Brcl1*^YS^ was significantly higher than that of YS. These results indicate that the mutant *Brcl1*^YS^ photosynthesis and resilience were enhanced.

### 2.4. Histological Analysis

**Figure 2:**
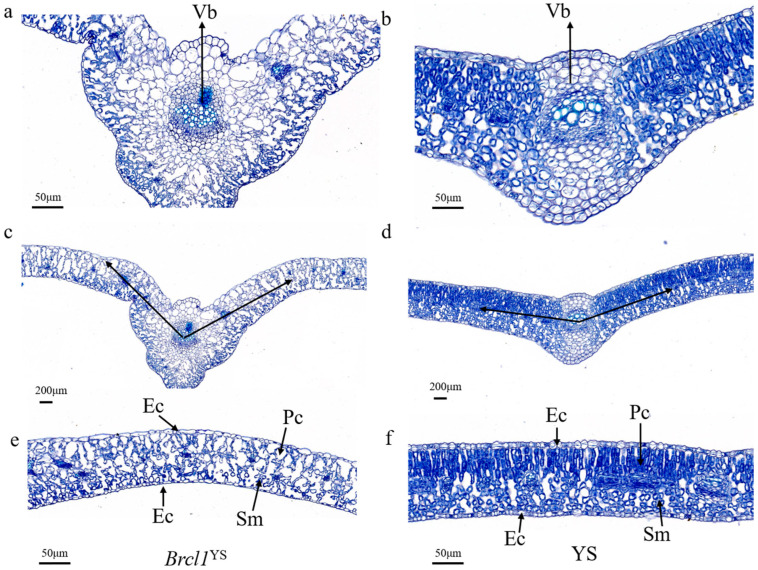
Cell observation of *Brcl1*^YS^ and YS. (**a**,**b**) The vascular bundle of *Brcl1*^YS^ and YS: scale bars = 50 μm; (**c**,**d**) Cross-section of leaf blade of *Brcl1*^YS^ and YS: scale bars = 200 μm, the arrow represents the angle between the leaf and the vascular bundle center; (**e**,**f**) The leaf cells of *Brcl1*^YS^ and YS: scale bars = 50 μm. Note: Vb—Vascular bundle; Ec—Epidermic cell; Pc—Palisade cell; Sm—spongy mesophyll.

The leaf of *Brcl1*^YS^ and YS were used to identify the cell morphology. The result showed that the vascular bundle cells of the mutant *Brcl1*^YS^ had a slight difference with YS (Figure 2a,b), but the *Brcl1*^YS^ leaves showed obvious curls from the vascular bundle center, and the curled degree of the leaves was about 103° (Figure 2c,d). Compared with the YS, the palisade cell and spongy mesophyll cells were smaller and arranged irregularly. Furthermore, the size of the epidermal cells was significantly different, as the cells were smaller in the curved surface but larger in the ventral surface in *Brcl1*^YS^ (Figure 2e,f).

### 2.5. Bulked Segregant RNA Sequencing (BSR-Seq) Analysis

**Figure 3:**
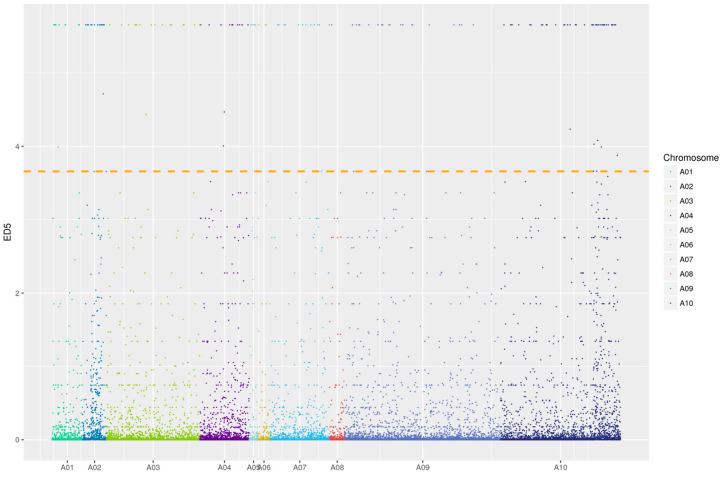
BSR-Seq analysis. The BSR-Seq based distribution of SNPs on chromosomes. The x-axis shows the 10 *B. rapa* chromosomes and the y-axis shows the ED^5^ values of the filtered SNPs; the dashed line is the threshold of the top 1%.

To identify the candidate interval for *Brcl1*^YS^, 45,494,770 and 46,962,128 high quality reads were mapped to the *B. rapa* reference genome V3.0 from the N-pool and C-pool, respectively. In total, compared with the reference genome, 197,180 SNPs and 210,789 InDels were identified in the BSR-seq data, respectively. Each of these variation loci were used to calculate the ED^5^. The results showed that the candidate region of the *Brcl1*^YS^ includes two regions, starting at 8,426,511 and ending at 9,253,907 on Chr. A02; the other region started at 16,103,255 and ended at 17,842,043 on Chr. A10 (Figure 3).

### 2.6. Fine Mapping of the Brcl1^YS^ Gene

According to the result of BSR-seq, we uniformly developed 10 pairs of InDel primers within two candidate intervals (Table S1).

**Figure 4:**
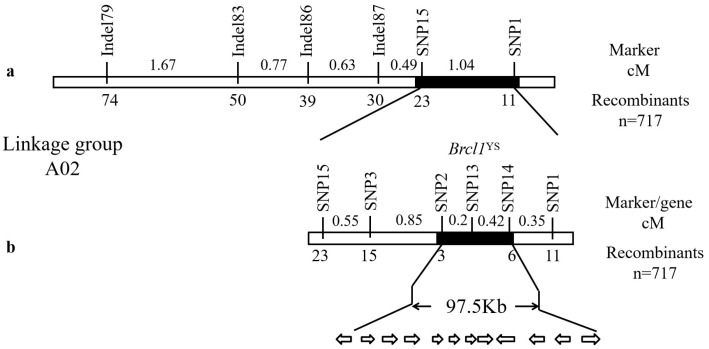
Fine mapping of the *Brcl1*^YS^ locus. (**a**) The *Brcl1*^YS^ locus was black-colored in an interval of 1.04 cM between SNP markers SNP1 and SNP15 on chromosome A02. (**b**) The candidate region was finely mapped in the physical interval of 97.5 kb between SNP2 and SNP14 markers.

Linkage analysis showed that the first interval in Chr. A02 (8,426,511–9,253,907) was linked to the *Brcl1*^YS^ locus. The F_2_ recessive homozygous, including 717 individuals, was used to narrow down the candidate interval. Indel79, Indel83, Indel86, Indel87, SNP15, SNP3 and SNP2 were located on one side of *Brcl1*^YS^, and SNP14 and SNP1 on the other side of *Brcl1*^YS^. SNP2 and SNP14 were tightly linked to *Brcl1*^YS^, with genetic distances of 0.20 cM and 0.42 cM, respectively. *Brcl1*^YS^ was mapped to a physical candidate interval of 97.5 kb (Figure 4).

### 2.7. Sequence Variations and Co-Segregation Verification of Candidate Genes

According to the *Brassica rapa* database (http://brassicadb.cn/#/Download/, accessed on 6 January 2021) and the homologous genes of *Arabidopsis* in TAIR (https://www.arabidopsis.org/, accessed on 6 January 2021), 12 genes (*BraA02g017010.3C*-*BraA02g017120.3C*) were located in the candidate interval in total (Table S2).

**Figure 5:**
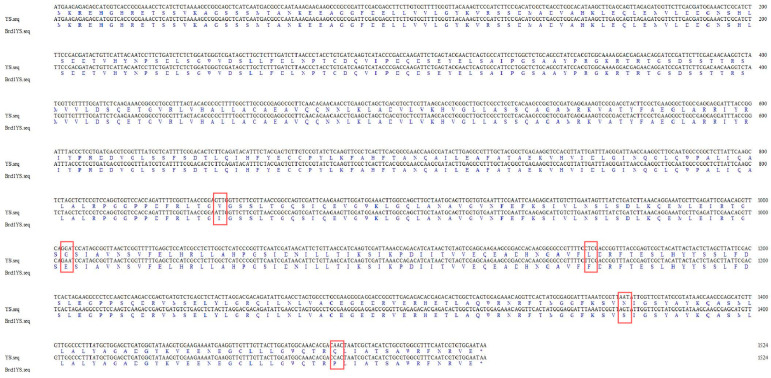
Candidate gene sequence analysis. The full length of *BraA02g017030.3C* between YS and *Brcl1*^YS^. The red boxes was five non-synonymous SNP mutations between YS and *Brcl1*^YS^, *: represents termination codon.

**Figure 6:**
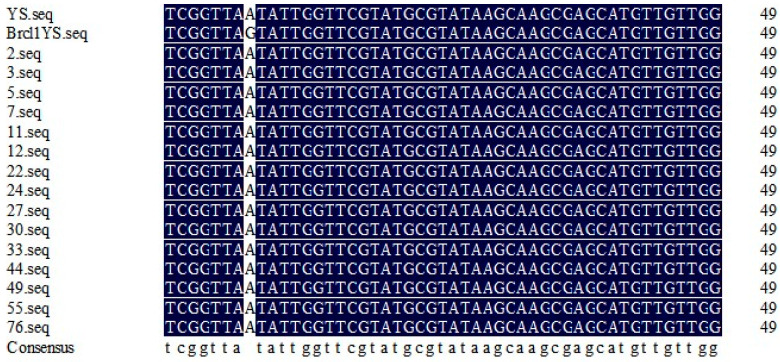
Candidate gene validation by co-separation of *Brcl1*^YS^. The mutant locus of YS, *Brcl1*^YS^ and recessive F_2_ individuals.

The results showed that the full length of *BraA02g017030.3C* was 1524 bp, including one exon and 508 amino acids. The *Brcl1*^YS^ promoter sequence remained unchanged when compared to YS, while the CDS sequence contained five non-synonymous SNP mutations. Asparagine changed to serine at position 1364 due to a G-base mutation to an A-base mutation that was consistent with the EMS mutation features (Figure 5). Co-separation primer found that this mutation exists in the fifteen recessive F_2_ individuals (Figure 6) thus we concluded that the *BraA02g017030.3C* was the most likely candidate gene.

### 2.8. Expression Pattern Analysis of BraA02g017030.3C

**Figure 7:**
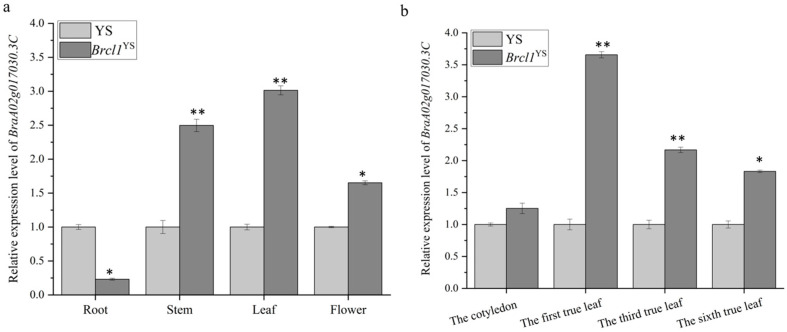
The expression pattern of *BraA02g017030.3C* in the two parents. (**a**) The expression level of *BraA02g017030.3C* in different organs (root, stem, leaf, flower) detected by qRT-PCR; (**b**) the expression level of *BraA02g017030.3C* in different leaf development periods (the cotyledon, the first true leaf, the third true leaf and the six true leaf) detected by qRT-PCR. The error bars represent the standard errors from three replications; * represents *p* < 0.05; ** represents *p* < 0.01.

To analyze the expression levels of *BraA02g017030.3C*, RNA was extracted from different organs (root, stem, leaf, flower) and leaves from different development periods (the cotyledon, the first, third and sixth true leaf) in YS and *Brcl1*^YS^, respectively. The results showed that the *BraA02g017030.3C* is expressed in each organ, with the highest expression observed in the first true leaf (Figure 7).

## 3. Discussion

The leaf is the main photosynthetic organ and its morphology is an important agronomic trait in breeding the ideal plant. Moderate leaf curling could enhance resistance to adversity while increasing the light area and reducing water loss [8]. Since the leaf is the edible part of *B. rapa*, leaf curling is a significant factor influencing the yield and quality [29]. Thus, the molecular basis of leaf curl might not only set a theory basis for the growth of *B. rapa* but also offer general insights into improving the resistance to abiotic stress.

Previous studies showed that appropriate leaf curling is beneficial to improve the photosynthetic efficiency of plants and increasing the effective accumulation of photosynthetic products, but severe leaf curling could inhibit plant growth [30,31]. *OsZHD1* and *OsACL1* were important genes for rice leaf curling. Severe leaf curling reduced the fertility rate and delayed the heading date. Rice *CLD1/SRL1* also modulated leaf rolling. The phenotypic characterization of the mutant found that leaf curling improved the content of chlorophyll [32,33]. In our study, we also detected the content of chlorophyll, Pn, Ci, E and Gs; the leaf curling mutant had higher chlorophyll and Pn, but Ci, E and Gs lower than normal leaf YS. The results indicate that the leaf curling trait locus may be useful to improve photosynthetic efficiency. The results are consistent with previous studies on *B. napus* [34,35]. Thus, breeding *B. rapa* varieties with moderate leaf curling could potentially improve the yield by improving the photosynthesis and stress resistance.

Functional and structural annotations showed that *BraA02g017030.3C* encodes the DELLA protein RGL1, which plays an inhibitor of GA transduction pathway and inhibited cell proliferation and expansion [25,36]. Recent utilization of CRISPR/Cas9 gene editing has resulted in the acquisition of a loss-of-function mutation in *BraRGL1* due to two amino acid changes in the GRAS domain. The flower bud differentiation and bolting time of *BraRGL1* mutants were significantly accelerated [37]. This finding is consistent with the observed phenotype of this material. However, whether there is functional redundancy of *RGL1* and whether it is related to leaf curling still needs to be verified by further experiments. Most studies show that *RGL1* is a significant factor in seed germination [38]. Meanwhile, the *AtWRKY45* and *AtWRKY75* transcription factors also interacted with *RGL1* to positively regulate age-triggered leaf senescence [39,40]. In addition, *RGL1* could combine with *AUXIN RESPONSE FACTOR 7* (*ARF7*) and *Aux/INDOLE-3-ACETIC ACID 9* (*IAA9*) to influence the IAA and GA signaling pathways, ultimately regulating cambial activity in poplar [41]. In *Arabidopsis*, a loss-of-function *rgl1* line had reduced GA_4_ expression and exhibited GA-independent activation of seed germination, leaf expansion, flowering, stem elongation, and floral development. Additionally, exogenous GA_3_ spraying could not rescue the phenotype [27]. In our study, we found that leaf curling, caused by changes in cells, may be influenced by GA or IAA signaling, and *Brcl1*^YS^ may play a role in this process. However, the function of *Brcl1*^YS^ in leaf curling has not been clear.

In summary, we employed BSR-seq based on F_2_ populations to identify the causal gene underlying *Brcl1*^YS^, which is responsible for curled leaves in *B. rapa*, as further validated using co-segregation analysis. The data on Pn, Ci, E, Gs and photosynthetic pigment content indicate that the mutation of *Brcl1*^YS^ has the potential to enhance the photosynthesis and stress resistance of *B. rapa* (Table 3). Sequence and expression pattern analysis of *BraA02g017030.3C* had a nonsynonymous mutation and was mainly expressed in the first true leaf. Therefore, *BraA02g017030.3C* is the most likely candidate gene. Combined with cell morphology observation, it is speculated that the loss of function of *Brcl1*^YS^ results in differences in cell development, ultimately leading to changes in leaf morphology.

## 4. Materials and Methods

### 4.1. Plant Materials and Phenotypic Evaluation

*Rapid Cycling Brassica rapa* (*RcBr*) is resistant to bolting and vernalization-dependent. The *B. rapa* curled leaf mutant1 (*Brcl1*) was derived from the *RcBr* with a flat blade by EMS mutagenesis. The seeds were provided by Scott Woody (University of Wisconsin). Subsequently, an individual from the cross between *Brcl1* and Yellow Sarson (YS) was selfed to develop the F_2_ segregating population. Ten individuals in the F_2_ population exhibiting a rolled leaf phenotype were selected for three successive backcrossing with the recurrent parent, YS, following one generation of selfing. Individuals exhibiting a rolled leaf phenotype were selected and designated as *Brcl1*^YS^, which were expected to be near-isogenic lines (NILs) for the recurrent parent, Yellow sarson. A cross between *Brcl1*^YS^ and YS was conducted to generate the new NIL-F_2_ population. The analysis was conducted on a total of 500 F_2_ individuals, which had been cultivated under greenhouse conditions since September 2018. Concomitantly, conventional linkage analysis was carried out on 1283 F_2_ individuals in March 2019. All plants were sown directly into 10 cm pots without additional vernalization. Phenotypic analysis was conducted at the Shenyang Agricultural University Experiment Station, Shenyang, China, at the following geographic coordinates: 41.8 °N, 123.4 °E.

A phenotypic investigation of the *Brcl1*^YS^ and YS was conducted utilizing digital display vernier calipers. Four indices were employed to assess the dissimilarities between *Brcl1*^YS^ and YS, including plant height, stem size, leaf width and leaf length. Once the plants had reached approximately 45 days of age, the mutant *Brcl1*^YS^ and YS, which exhibited similar growth trends, were selected for further analysis. The mean of 10 individuals was employed to ascertain the disparity in phenotypic data via Student’s *t*-test for each index, utilizing SPSS v17.0 (IBM Corp., Armonk, NY, USA).

### 4.2. Genetic Analysis

To ascertain the genetics of curled leaves in *B. rapa*, phenotypic characterization was carried out for each generation (*Brcl1*^YS^, YS, F_1_, F_2_), and the segregation ratios of the F_2_ populations were determined by using the Chi-square test [42].

### 4.3. Photosynthetic Index and Chlorophyll Content Determination

To determine the growth differences between *Brcl1*^YS^ and YS, measurements were made of the photosynthetic rate (Pn), the intercellular CO_2_ concentration (Ci), the transpiration rate (E) and stomatal conductance (Gs) using a Li-6400 portable photosynthesis system (LI-COR Biosciences, Lincoln, NE, USA). On a sunny day between 08:00–12:00 h, the photosynthetic index for *Brcl1*^YS^ and YS was measured at the same growth stage after the plant’s fourth true leaf had fully formed. Photosynthetic indices were measured using a modified method [43]. There were three duplicates carried out, each including six plants. Ten *Brcl1*^YS^ and YS leaves were mixed with the solution using a 0.5 cm diameter punch and left in the dark for 24 h. The method of chlorophyll and carotenoid contents were conducted according to previous studies [44].

### 4.4. Paraffin Section

Before being paraffin sectioned, fresh leaves of *Brcl1*^YS^ and YS were fixed for 24 h in formalin-glacial acetic acid (FAA). The leaves were then pumped outside to dry naturally. The samples were dehydrated for two hours using varying alcohol concentrations (30–95%). Then, overnight at 4 °C, they were incubated in 100% alcohol before being penetrated with xylene. Then, paraffin embedding and sectioning were conducted using a microtome (LeicaRM2016, Leica, Wetzlar, Germany). Using an optical microscope (Nikon ECLIPSE 80i, Tokyo, Japan), the stem and leaf shapes were observed. Using the NIS-Element SF3.2 software’s capture option, the photo was taken, the adjusted image was chosen, and it was saved (Nikon, Tokyo, Japan).

### 4.5. BSR-Seq Analysis

In order to perform BSR-Seq analysis, two pools were formed by mixing equal amounts of RNA from 50 normal plants (N pool) and 50 curled plants (C pool), based on the phenotypic scores of 500 F_2_ individuals recorded in September 2018.

Following the construction of the cDNA libraries, sequencing was performed using the Illumina HiSeq 2500 platform (Illumina, San Diego, CA, USA) and the data analysis was handled by Personalbio in Shanghai, China. Trimmomatic v0.30 (Illumina, CA, USA) was used to eliminate low-quality bases in order to produce clean data, ensuring data accuracy [45]. Using Hisat2 (Johns Hopkins University, Baltimore, MD, USA), the clean data were compared to the *B. rapa* reference genome V3.0 (BRAD; http://brassicadb.cn/#/Download/, accessed on 12 March 2019) [46]. Samtools v0.1.18 and Bcftools v0.1.19 (Trust Sanger Institute, Cambridgeshire, UK) were used to detect single nucleotide variation (SNV) [47,48]. Based on an mpileup file produced by samtools v0.1.18 (Trust Sanger Institute, Cambridgeshire, UK) for BSR-seq, the Euclidean distance (ED) value was computed. The ED value of each unique SNV site was raised to the power of five, or ED^5^, in order to remove background noise [49]. The top 1% of ED^5^ values were used as a threshold in the screening process to identify the candidate area for the curled leaf phenotype. This was then compared with multiple SNV sites spread across various chromosomes.

### 4.6. DNA Extraction, Marker Development, Linkage Map Construction

Total DNA was extracted using the CTAB technique with a few minor modifications [50]. The amplification and PCR reaction volumes followed the earlier instructions [44]. Primer Premier 5.0 software (Premier Biosoft, San Francisco, CA, USA) was used to construct the primers for the InDel markers. The BSR-seq database was used to identify sequence variations in the candidate interval, which were utilized to design SNP or InDel markers. Join Map 4.0 was used to create a genetic linkage map [51]. Using Kosambi’s function [52], the genetic map distances (cM) were derived from the recombination frequencies. Table S1 displays the details of the primer.

### 4.7. Candidate Gene Prediction and Co-Separation Verification

The *Brassica rapa* database (BRAD; http://brassicadb.cn/#/Download/, accessed on 6 January 2021) and the *Arabidopsis* Information Resource (TAIR; https://www.arabidopsis.org/, accessed on 6 January 2021) provided the gene information found in the candidate interval. The exact primers for co-separation and full-length sequencing amplification were designed using Primer Premier 5.0 (Table S1). The PCR products were purified using a Gel Extraction Kit (CWBIO, Beijing, China) and then ligated into a pGEM-T Easy Vector (Promega, Madison, WI, USA) for sequencing at Sangon Biotech (Shanghai, China). Using the DNAMAN program (Lynnon Biosoft, San Ramon, CA, USA), the sequences were aligned. For co-separation verification, fifteen F_2_ homozygous recessive individuals were used.

### 4.8. The Expression Pattern of the Candidate Gene

The candidate gene’s level of expression was assessed using qRT-PCR. The different organs (root, stem, leaf, flower) and leaves from different development periods (the cotyledon, the first true leaf, third true leaf and sixth true leaf) were used to extract the total RNA of *Brcl1*^YS^ and YS using an RNA extraction kit (Aidlab Biotechnologies Co., Ltd., Beijing, China). The RNA was then subjected to reverse transcription to produce cDNA. Quantitative real-time PCR (qPCR) was performed using the cDNA template in a 25 µL reaction volume [44]. The relative expression level was analyzed with the 2^−ΔΔCT^ technique [53]. The cycle threshold (Ct) values were determined by averaging three separate biological replicates. Every sample underwent three separate technical replicate evaluations. The QuantStudio™ Real-Time PCR Software V1.3 (ABI, Los Angeles, CA, USA) was utilized to analyze the data. Gene-specific primers were designed using Primer Premier 5.0 (Table S1). The *Actin* gene (encoding beta actin) formed the reference gene [54].

## 5. Conclusions

*BraA02g017030.3C* was the most promising gene conferred to *Brcl1*^YS^, which is homologous with *Arabidopsis RGL1*, belonging to DELLA protein and involved in the GA signal pathway. These results could facilitate our understanding of the mechanisms underlying leaf morphogenesis and provide a genetic resource for *B. rapa* improvement.

## Figures and Tables

**Table 1:** Measurements of phenotype of YS and *Brcl1*^YS^.

	Plant Height (cm)	Stem Width (cm)	Leaf Length (cm)	Leaf Width (cm)
YS	8.97 ± 0.76	0.74 ± 0.02	8.38 ± 0.53	5.38 ± 0.22
*Brcl1* ^YS^	4.98 ± 0.44 **	0.62 ± 0.06 **	6.13 ± 0.26 **	3.35 ± 0.19 **

** indicates significant difference at the 0.01 level.

**Table 2:** Genetic analysis of *Brcl1*^YS^.

Population Employed	Pedigree	Total Plants	Curled Leaves	Normal Leaves	Expected Ratio	X^2^
*Brcl1* ^YS^		50	50	0		
YS		50	0	50		
F_1_	YS×*Brcl1*^YS^	50	50	0		
RF_1_	*Brcl1*^YS^×YS	50	50	0		
F_2_-A	(YS×*Brcl1*^YS^) ⊗	500	381	119	3:1	0.40
F_2_-B	(YS×*Brcl1*^YS^) ⊗	1283	953	330	3:1	0.35

⊗ represent selfed.

**Table 3:** Photosynthetic index determination of YS and *Brcl1*^YS^.

	Pn (μmol m^−2^ s^−1^)	E (mmol m^−2^ s^−1^)	Ci (μmol mol^−1^)	Gs (mmol m^−2^ s^−1^)	Chla	Chlb	Chl	Car
					(mg g^−1^ FW)			
YS	10.75 ± 0.77	5.13 ± 0.87	351.88 ± 27.01	0.35 ± 0.73	1.57 ± 0.80	0.52 ± 0.27	2.09 ± 0.34	0.20 ± 0.07
*Brcl1* ^YS^	12.69 ± 0.74 *	4.05 ± 0.86 *	315.26 ± 30.29 **	0.23 ± 0.11 *	1.85 ± 0.42 *	0.79 ± 0.18 *	2.34 ± 0.59 *	0.44 ± 0.14 *

* indicate significant difference at the 0.05 level; ** indicate significant difference at the 0.01 level.

## Data Availability

The data supporting the results are included in this article. Additional relevant materials are available upon reasonable request from the corresponding author. The raw data from Bulked segregant RNA sequencing have been deposited at the NCBI Sequence Read Archive (SRA) repository under the accession numbers SRR29689162 and SRR29689163.

## Supplementary Materials

The following supporting information can be downloaded at: https://www.mdpi.com/article/10.3390/ijms26020732/s1.
